# Health outcome of older hospitalized patients in internal medicine environments evaluated by Identification of Seniors at Risk (ISAR) screening and geriatric assessment

**DOI:** 10.1186/s12877-019-1239-3

**Published:** 2019-08-14

**Authors:** Anne-Carina Scharf, Janine Gronewold, Christian Dahlmann, Jeanina Schlitzer, Andreas Kribben, Guido Gerken, Tienush Rassaf, Christoph Kleinschnitz, Richard Dodel, Helmut Frohnhofen, Dirk M. Hermann

**Affiliations:** 1 Department of Neurology, University Hospital Essen, University of Duisburg-Essen, Hufelandstraße 55, 45147 Essen, Germany; 2 Nursing Headquarters, University Hospital Essen, University of Duisburg-Essen, Hufelandstraße 55, 45147 Essen, Germany; 3 Department of Nephrology, Geriatric and Internal Medicine, Alfried Krupp Hospital Ruettenscheid-Essen, Alfried-Krupp-Straße 21, 45131 Essen, Germany; 4 Department of Nephrology, University Hospital Essen, University Duisburg-Essen, Hufelandstraße 55, 45147 Essen, Germany; 5 Department of Gastroenterology and Hepatology, Faculty of Medicine, University Hospital Essen, University of Duisburg-Essen, Hufelandstraße 55, 45147 Essen, Germany; 6 Department of Cardiology and Vascular Medicine, West German Heart and Vascular Center, University Hospital Essen, Hufelandstraße 55, 45147 Essen, Germany; 7 Department of Geriatrics, University Hospital Essen, Germaniastraße 1-3, 45356 Essen, Germany; 8 Faculty of Health, Department of Medicine, University Witten-Herdecke, Witten, Germany

**Keywords:** ISAR, CGA, Older in-patients, Risk screening, Geriatrics, Internal medicine, Cutoff, Sensitivity, Specificity

## Abstract

**Background:**

Hospitals are in need of valid and economic screening and assessment tools that help identifying older patients at risk for complications which require intensified support during their hospital stay.

**Methods:**

Five hundred forty-seven internal medicine in-patients (mean age 78.14 ± 5.96 years; 54.7% males) prospectively received Identification of Seniors at Risk (ISAR) screening. If screening results were positive (ISAR score ≥ 2), a comprehensive geriatric assessment (CGA) was performed. We explored sensitivity and specificity of different ISAR and CGA cutoffs. Further, we analyzed the risk of falls and how patients got discharged from hospital.

**Results:**

ISAR+/CGA abnormal patients spent more days in hospital (16.1 ± 14.5), received more nursing hours per day (3.0 ± 2.3), more hours of physiotherapy during their hospital stay (2.2 ± 3.2), and had more falls (10.1%) compared to ISAR+/CGA normal (10.9 ± 12.3, 2.0 ± 1.2, 1.2 ± 4.3, and 2.8%, respectively, all *p* ≤ 0.016) and ISAR- (9.6 ± 11.5, 2.3 ± 4.5, 0.7 ± 2.0, and 2.2%, respectively, all *p* ≤ 0.002) patients. ISAR+/CGA abnormal patients terminated their treatment regularly with being discharged back home less often (59.6%) compared to ISAR+/CGA normal (78.5%, *p* = 0.002) and ISAR- (78.2%, *p* = 0.056) patients. ISAR cutoff≥2 and CGA defined as abnormal in case of impairment of ADL plus another CGA domain achieved best sensitivity/specificity.

**Conclusions:**

Abnormal geriatric risk screening and assessment are associated with longer hospital stay and higher amount of nursing and physiotherapy during hospital stay, greater risk of falling, and a lower percentage of successfully terminated treatment in older in-patients.

## Background

Due to ongoing demographic aging, hospitals face a constantly rising number of older patients with multimorbidity [1–3]. Although older people represent a challenge for the hospital setting, excellent medical attendance and high-quality care should be ensured. The use of screening tools allows for the identification of older patients at increased risk for poor health outcomes. Worldwide, geriatric societies demand the implementation of screening tools for the early identification of patients at increased risk for poor health outcomes [4–6]. The Identification of Seniors at Risk (ISAR) screening is one of the most commonly used tools with high sensitivity for the prediction of poor health outcomes in older patients entering emergency departments [7]. Created as a screening tool, ISAR requires a second-step diagnostic tool for patients with positive screening results. Comprehensive geriatric assessment (CGA) that evaluates impairments of activities of daily living (ADL), mobility, cognition, and mood as well as comorbidities is usually performed on patients with a positive screening result. Despite being only a diagnostic tool and not an intervention strategy, CGA preceded by ISAR screening has already been shown to reduce the risk for poor health outcomes in older patients attending emergency departments [8]. It further improves postoperative outcomes (mortality, delirium, and length of hospital stay) in older patients with colorectal carcinoma undergoing elective resection [9]. In addition, the probability of living at home one year after being released from hospital was about 16% higher in geriatric hospitalized patients undergoing CGA compared to those who received the usual care [1]. These data suggest that CGA leads to an improvement of individual patient health outcomes while lowering the costs associated with diseases, nursing, and health care [10].

Besides emergency department patients and in-patients undergoing surgery, patients admitted to internal medicine departments also challenge healthcare professionals to identify needs and risks for poor health outcomes. Since ISAR was originally designed as a screening tool in emergency departments, we herein extended ISAR’s utility and used the ISAR for defining the health outcome of older hospitalized internal medicine patients. We sought to determine the association between ISAR screening (with CGA if positive on screening) and length of stay, nursing and physiotherapy hours, risk of falls, and discharge disposition among older adults admitted to internal medicine departments. In sensitivity analyses, we explored the sensitivity and specificity of different ISAR and CGA cutoffs for identifying outcomes among older adults admitted to internal medicine departments.

## Methods

### Study cohort

Patients admitted to internal medicine wards of the University Hospital Essen via emergency departments or as selective inpatient admission or being transferred from another ward or hospital from July 2015 to February 2017 were included in the present study if they received ISAR screening and were (a) ≥75 years of age in the Department of Gastroenterology and Hepatology and the Department of Cardiology and Angiology or (b) aged ≥65 years in the Department of Nephrology. Nephrological patients were included based on a younger age criterion as their biological age appears to be higher than their chronological age [11, 12]. We decided to apply ISAR in these three departments because these departments cover all significant geriatric patient groups in our University Hospital within the internal medicine specialty. ISAR screenings were conducted by the nursing staff on admission and were only missed when there was a lack of time, language barriers or incompliant patients. Those who were not given ISAR were excluded from any further analyses. In case of a positive ISAR screening result, CGA was performed by a geriatric liaison service usually the day following ISAR screening and 3 days after admission the latest. The geriatric liaison service of the University Hospital Essen consisted of a geriatrician, an occupational therapist, and a psychologist. In all subjects, patient histories involving information about comorbidities and vascular risk factors were taken from the electronical Hospital Information System Cerner medico. The study was approved by the ethics committee of the University Duisburg-Essen and need for consent was waived.

### Measurement methods

#### ISAR

In this study, we utilized a version of ISAR by Warburton [13] validated for patients aged ≥75 years which was a modification of the original test by McCusker et al. [14]. The ISAR consists of six items, each being a simple yes-no question about the following domains: Premorbid functional dependence, acute change in functional dependence within the last 24 h, recent hospitalization within the last 6 months, visual impairment, impaired memory and polypharmacy (≥6 medications). The ISAR score ranges from 0 to 6 points, with a cutoff ≥2 interpreted as positive (abbreviated as ISAR+) and indicating increased risk for poor health outcomes.

#### CGA

Since there is an ongoing discussion on which geriatric impairments are associated with deteriorated health condition, we analyzed different definitions of an abnormal CGA, which included the Barthel index for the assessment of impairment of ADL [15, 16], the Timed Up & Go [17] and the Tinetti Mobility Test [18] measuring impairment of mobility, the Mini-Mental State Examination Test (MMSE) [19] and the Clock-Drawing Test [20] assessing impairment of cognition, and the Geriatric Depression Scale (GDS) [21, 22] for the assessment of signs of depression. The Barthel index is a questionnaire assessing daily competences in which patients can reach a maximum score of 100 and scores < 90 are interpreted as abnormal [23]. Mobility was rated as impaired if Timed Up & Go was ≥20 s [24] or if patients had scores < 20 in the Tinetti Mobility Test [25]. Impaired cognition was defined as MMSE ≤27 [26, 27] or Clock-Drawing Test ≥3 [20] and a GDS score ≥ 6 [28] was interpreted as a sign of depression. If not noted differently, we interpreted CGA as abnormal in this study if Barthel index and one other domain (mobility, cognition, or signs of depression) were impaired (abbreviated as ISAR+/CGA abnormal) as suggested by Campbell et al. [29].

#### Health outcome variables

As indicators for poor health outcomes, we analyzed length of hospital stay, nursing hours per day, physiotherapy workload, falls during the hospitals stay, and type of discharge from hospital using data obtained from the electronical Hospital Information System Cerner medico. Nursing hours were operationalized using the “Leistungserfassung in der Pflege”, a scientifically valid tool documenting nursing workload (for further details see Gronewold et al. [30]).

We also reported the patients’ type of hospital discharge. We classified if patients terminated their treatment regularly with being discharged back home or being transferred to further medical care. Further medical care was split into planned or unplanned subsequent readmission, transfer to other hospitals, and transfer to rehabilitation or nursing institution. We also indicated whether treatment was terminated against medical advice and if the patients died while in hospital.

### Statistical analysis

Continuous data are presented as mean ± SD values, categorical data as counts (%). Comparisons between negative ISAR screening (ISAR-), ISAR+/CGA normal and ISAR+/CGA abnormal groups regarding demographic data, risk factors and comorbidities, number of falls, type of discharge, length of hospital stay, and nursing and physiotherapy hours were done with (1) one-way ANOVA followed by Games Howell post-hoc tests for normally distributed continuous data (age), (2) Kruskal-Wallis tests and post-hoc Mann-Whitney u test (corrected for multiple comparisons where needed) for not normally distributed continuous data (length of hospital stay, hours of nursing per day and physiotherapy during hospital stay) and (3) Pearson’s chi-square or Fisher’s exact tests for categorical data.

Since CGA is costly and time-consuming, screening instruments with high sensitivity and specificity for the identification of patients needing further risk assessment are needed. Thus, we analyzed the sensitivity and specificity of different ISAR cutoffs for the prediction of length of hospital stay (≥7 days), nursing (above median) and receiving physiotherapy (yes/no). Further, we used receiver operating characteristics (ROC) including the area under the curve (AUC) and confidence intervals as well as Youden’s J statistics [sensitivity + specificity − 1]. Since there is no agreement on which tests a CGA should include and when a CGA should be interpreted as abnormal, we analyzed different definitions of an abnormal CGA. In line with published suggestions [31, 32], abnormal CGA was first defined as significant impairment of ADL combined with impairment of one other CGA test domain (cognition, mobility or signs of depression). In sensitivity/specificity analyses, we also evaluated alternative definitions. Again, we used these different alternative definitions of abnormal CGA for the prediction of length of hospital stay (≥7 days), nursing (above median) and receiving physiotherapy (yes/no) and calculated the Youden’s J statistics.

*P* values ≤0.05 indicate statistical significance and are shown in bold in the tables. All statistics were performed using Statistical Packing for Social Science 22 (SPSS 22) for Windows (SPSS, Chicago, IL, U.S.A.).

## Results

### Study cohort

#### Demographic and medical data

Of 1329 patients fulfilling the above inclusion criteria (76.62 ± 6.3 years, 55.7% males), 547 patients (41.2%) received ISAR screenings. Patients receiving ISAR screenings were 78.1 ± 6.0 years old (54.7% males) and stayed in hospital for 11.22 ± 13.9 days, patients not receiving ISAR screening were slightly younger (75.66 ± 6.40 years, 56.5% males) and stayed in hospital considerably shorter for 8.96 ± 11.9 days. Of the 547 screened patients, 318 (58.1%) had a positive screening result (ISAR score ≥ 2). Of these patients, 242 (76.1%) received a subsequent CGA, which was abnormal in 97 (40.1%) patients. The 76 ISAR+ patients who did not receive CGA did not differ significantly from ISAR+ patients who received CGA on patients’ characteristics in Table 1. Reasons for not performing a CGA despite positive screening results were transfer to another hospital or ward, discharge, foreign-language barriers or incompliance of patients.

**Table 1:** Characteristics of the total cohort also split by ISAR and CGA results

	Total (*n* = 547)	ISAR- (*n* = 229; 41.9%)	ISAR+/CGA normal (*n* = 145; 26.5%)	ISAR+/CGA abnormal (*n* = 97; 17.7%)	*p*-value ISAR+/CGA normal vs ISAR-	*p*-value ISAR+/CGA abnormal vs ISAR-	*p*-value ISAR+/CGA abnormal vs ISAR+/CGA normal
Age (years)	78.1 ± 6.0	77.9 ± 5.4	77.0 ± 5.9	80.5 ± 6.5	0.259	**0.001**	**<0.001**
Sex (male)	299 (54.7)	122 (53.3)	96 (66.2)	43 (44.3)	**0.013**	0.118	**<0.001**
Anemia	152 (27.8)	55 (24.0)	51 (35.2)	29 (29.9)	**0.019**	0.336	0.318
Chronic kidney disease	240 (43.9)	86 (37.6)	75 (51.7)	43 (44.3)	**0.007**	0.327	0.194
Heart failure	106 (19.4)	39 (17.0)	35 (24.1)	16 (16.5)	0.140	0.999	0.263
Coronary heart disease	213 (38.9)	90 (39.3)	51 (35.2)	45 (46.4)	0.511	0.329	0.142
Atrial fibrillation	174 (31.8)	62 (27.1)	47 (32.4)	37 (38.1)	0.293	**0.050**	0.412
Other cardiac arrhythmias	70 (12.8)	23 (10.0)	18 (12.4)	17 (17.5)	0.498	0.097	0.354
Valve insufficiency	196 (35.8)	78 (34.1)	53 (36.6)	39 (40.2)	0.738	0.213	0.423
Chronic obstructive pulmonary disease	69 (12.6)	25 (10.9)	17 (11.7)	15 (15.5)	0.867	0.277	0.448
Peripheral artery disease	76 (13.9)	27 (11.8)	22 (15.2)	21 (21.6)	0.348	**0.040**	0.237
Arterial hypertension	430 (78.6)	175 (76.4)	113 (77.9)	80 (82.5)	0.704	0.311	0.626
Diabetes	171 (31.3)	71 (31.0)	45 (31.0)	33 (34.0)	0.999	0.606	0.676
Hyperlipoproteinemia	269 (49.2)	96 (41.9)	79 (54.5)	55 (56.7)	**0.025**	**0.011**	0.694
Nicotine abuse	82 (15.0)	34 (14.8)	25 (17.2)	11 (11.3)	0.561	0.484	0.202
Obesity	116 (21.2)	46 (20.1)	29 (20.0)	23 (23.7)	0.999	0.463	0.427
History of myocardial infarction	56 (10.2)	23 (10.0)	9 (6.2)	13 (13.4)	0.255	0.443	0.073
History of pulmonary embolism	11 (2.0)	2 (0.9)	4 (2.8)	3 (3.1)	0.378	0.070	0.447
History of stroke	56 (10.2)	14 (6.1)	16 (11.0)	15 (15.5)	0.116	**0.006**	0.255
History of thrombosis	49 (9.0)	19 (8.3)	10 (6.9)	12 (12.4)	0.695	0.306	0.179
Hyperthyroidism	24 (4.4)	15 (6.6)	1 (0.7)	5 (5.2)	**0.007**	0.802	**0.042**
Hypothyroidism	84 (15.4)	30 (13.1)	30 (20.7)	11 (11.3)	0.081	0.859	0.118
Dementia	37 (6.8)	4 (1.7)	9 (6.2)	16 (16.5)	0.067	**<0.001**	**0.005**
Alcohol abuse	19 (3.5)	6 (2.6)	7 (4.8)	2 (2.1)	0.261	0.999	0.317
Depression	30 (5.5)	9 (3.9)	5 (3.4)	7 (7.2)	0.999	0.265	0.236
Anxiety disorder	2 (0.4)	1 (0.4)	0 (0.0)	0 (0.0)	0.999	0.999	0.999
Parkinson’s disease	6 (1.1)	3 (1.3)	2 (1.4)	0 (0.0)	0.999	0.557	0.515
Polyneuropathy	40 (7.3)	13 (5.7)	10 (6.9)	14 (14.4)	0.825	**0.009**	**0.028**
Cancer	215 (39.3)	89 (38.9)	73 (50.3)	21 (21.6)	**0.041**	**0.003**	**<0.001**
Cataract	33 (6.0)	8 (3.5)	13 (9.0)	6 (6.2)	**0.036**	0.371	0.472
Presbycusis	21 (3.8)	8 (3.5)	4 (2.8)	7 (7.2)	0.773	0.161	0.128
Anal incontinence	3 (0.5)	1 (0.4)	2 (1.4)	0 (0.0)	0.562	0.999	0.515
Urinary incontinence	4 (0.7)	1 (0.4)	0 (0.0)	2 (2.1)	0.999	0.218	0.165
Pressure ulcers	24 (4.4)	1 (0.4)	4 (2.8)	14 (14.4)	0.075	**<0.001**	**0.002**
Rheumatism	24 (4.4)	11 (4.8)	5 (3.4)	4 (4.1)	0.609	0.999	0.999

CGA, comprehensive geriatric assessment; ISAR, Identification of Seniors at Risk; ISAR+, positive ISAR screening (score ≥ 2); ISAR-, negative ISAR screening (score < 2); CGA abnormal, impairment of ADL plus another domain of the CGA. In 318 ISAR+ patients, 242 CGAs were performed (76 missing due to transfer, discharge, foreign-language or incompliance of patients). Boldface values were significant at *p* <=0.05

Demographic and medical data including comorbidities and risk factors for the total cohort and split by ISAR and CGA results are shown in Table 1. Various diseases were coded as main medical diagnoses leading to hospital admission (Table 2). Nearly 80% of the cohort suffered from comorbid arterial hypertension and about half of the cohort suffered from hyperlipoproteinemia. Clinical diagnosis of dementia and depression known before the CGA had a rather low prevalence of 7 and 6% in the total cohort.

**Table 2:** Main medical diagnosis leading to hospital admission of the total cohort also split by ISAR and CGA results

	Total (*n* = 547)	ISAR- (*n* = 229)	ISAR+/CGA normal (*n* = 145)	ISAR+/CGA abnormal (*n* = 97)
Liver cancer	71 (13.0)	30 (13.1)	25 (17.2)	5 (5.2)
Renal transplantation	55 (10.1)	15 (6.5)	24 (16.6)	9 (9.3)
CKD (not dialysis-dependent)	38 (6.9)	17(7.4)	10 (6.9)	4 (4.3)
Peripheral artery disease	30 (5.5)	16 (7.0)	7 (4.8)	6 (6.2)
Aortic valve stenosis	25 (4.6)	6 (2.6)	4 (2.8)	9 (9.3)
CKD requiring dialysis	21 (3.8)	11 (4.8)	8 (5.5)	0 (0.0)
Arterial hypertension	19 (3.5)	12 (5.2)	3 (2.1)	2 (2.1)
Acute renal failure	19 (3.5)	7 (3.1)	3 (2.1)	5 (5.2)
Heart failure	17 (3.1)	6 (2.6)	3 (2.1)	5 (5.2)
Neoplasia of the gastrointestinal tract	14 (2.6)	9 (3.9)	4 (2.8)	0 (0.0)
Cholangiocarcinoma	13 (2.4)	6 (2.6)	3 (2.1)	1 (1.0)
Liver cirrhosis	10 (1.8)	3 (1.3)	5 (3.4)	1 (1.0)
Cholangitis	8 (1.5)	4 (1.7)	1 (0.7)	1 (1.0)
ST-segment elevation myocardial infarction	8 (1.5)	3 (1.3)	1 (0.7)	2 (2.1)
Aneurysm	8 (1.5)	8 (3.5)	0 (0.0)	0 (0.0)
Angina pectoris	8 (1.5)	5 (2.2)	1 (0.7)	0 (0.0)
Vasculitides	8 (1.5)	1 (0.4)	4 (2.8)	1 (1.0)
Infectious diseases	7 (1.3)	0 (0.0)	3 (2.1)	3 (3.1)
Diverticulosis	7 (1.3)	3 (1.3)	1 (0.7)	3 (3.1)
Non-ST-segment elevation myocardial infarction	7 (1.3)	4 (1.7)	0 (0.0)	2 (2.1)
Mitral valve stenosis or insufficiency	7 (1.3)	2 (0.9)	1 (0.7)	4 (4.3)
Bile duct strictures	6 (1.1)	3 (1.3)	1 (0.7)	0 (0.0)
Aortic dissection	6 (1.1)	2 (0.9)	1 (0.7)	2 (2.1)
Cholelithiasis	5 (0.9)	3 (1.3)	1 (0.7)	1 (1.0)
Gastrointestinal bleeding	5 (0.9)	4 (1.7)	0 (0.0)	0 (0.0)
Pancreatic cysts	5 (0.9)	3 (1.3)	1 (0.7)	0 (0.0)
Urinary tract infection	4 (0.8)	0 (0.0)	1 (0.7)	2 (2.1)

Data are total numbers complemented in brackets by frequencies. CGA, comprehensive geriatric assessment; ISAR, Identification of Seniors at Risk; ISAR+, positive ISAR screening (score ≥ 2); ISAR-, negative ISAR screening (score < 2); CGA abnormal, impairment of ADL plus another domain of the CGA; CKD, chronic kidney disease

#### ISAR screening and CGA results

**Fig. 1:**
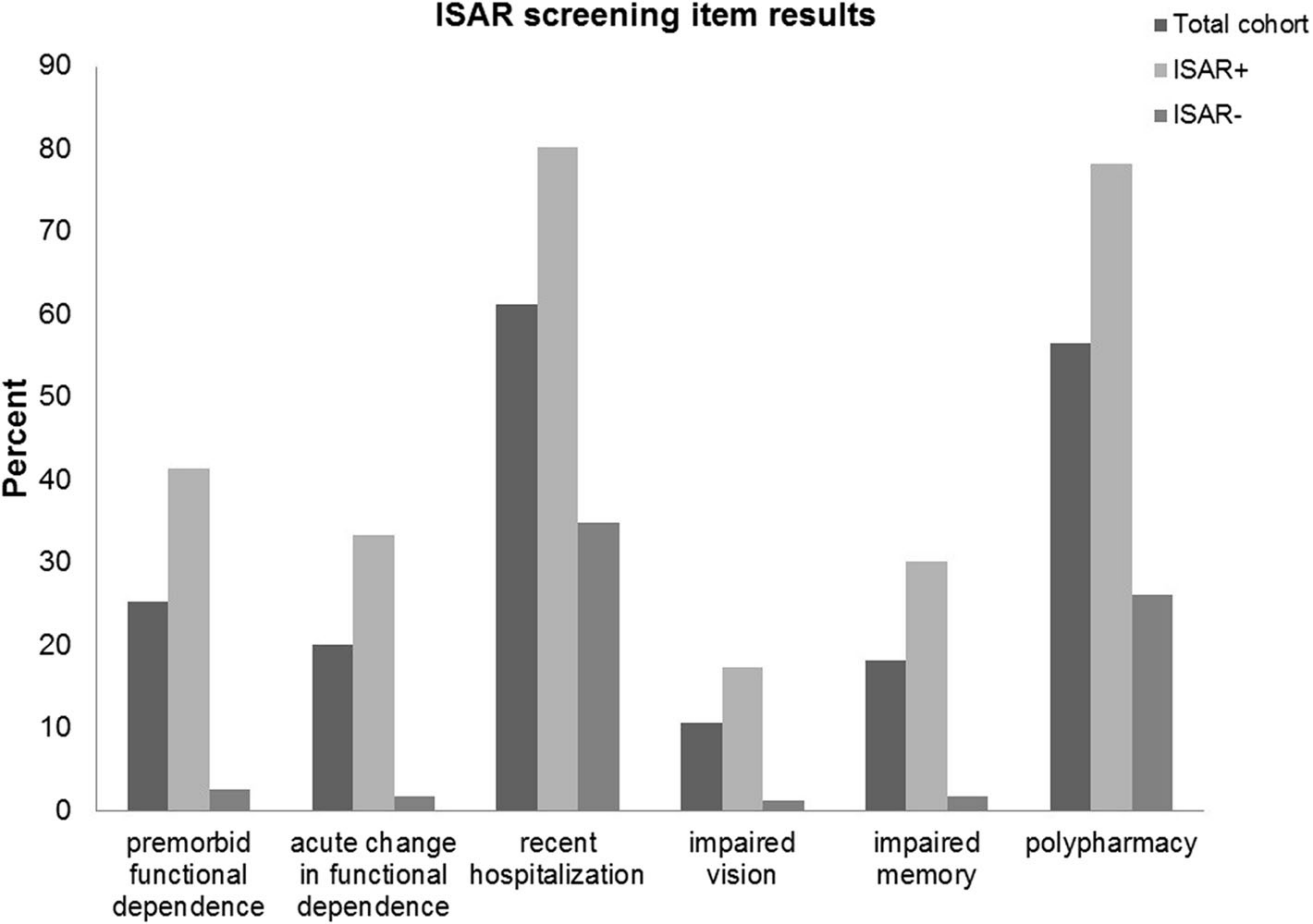
“Identification of Seniors at Risk” items for total cohort and separately for patients with positive and negative ISAR screening result. ISAR, Identification of Seniors at Risk; ISAR+, positive ISAR screening (score ≥ 2); ISAR-, negative ISAR screening (score < 2)

More than half of the total cohort showed recent hospitalization (61.2%) and polypharmacy (56.5%) whereas premorbid functional dependence (25.2%), acute change in functional dependence (20.1%), impaired vision (10.6%) and impaired memory (18.3%) were reported less often (Fig. 1). Looking at the domains affected, 47.3% of the total cohort had impaired ADL, 35.6% impaired mobility, 54.4% impaired cognition, and 11.6% showed signs of depression. Interestingly, even in patients without prior dementia diagnosis, 51.9% had impaired cognition in CGA and in patients without prior diagnosis of depression we found signs of depression in 11.4% in CGA.

#### Sensitivity and specificity analyses of different ISAR cutoffs for the prediction of length of hospital stay, nursing hours and physiotherapy

**Fig. 2:**
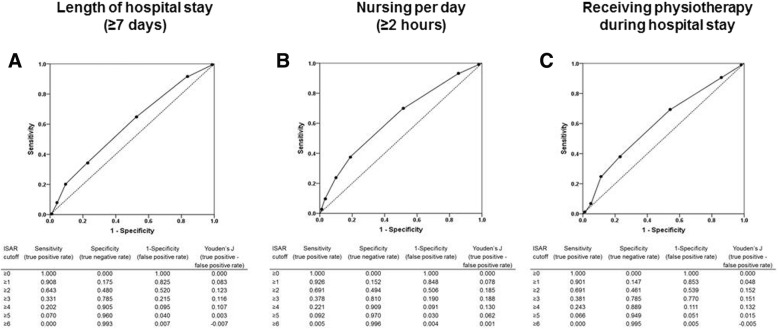
Receiver operating characteristics of ISAR score for the prediction of **a**) hospital stay ≥7 days, **b**) nursing hours above the median in the cohort (≥2 h) and **c**) receiving physiotherapy during hospital stay. ISAR, Identification of Seniors at Risk

The ROC results for the ISAR screening for the prediction of a hospital stay ≥7 days, i.e., the precondition for geriatric rehabilitation in several countries including Germany, revealed an AUC = 0.593 (95% CI = 0.545–0.640), indicating poor discriminating ability of ISAR. Yet, compared to other cutoffs, the Youden’s J index still revealed best performance for the ≥2 cutoff as suggested in the literature [33], with a true positive rate (sensitivity) of 0.643 and false positive rate (1-specificity) of 0.520 (Fig. 2).

The analysis of the predictive value for the ISAR in predicting nursing hours split by the median (≥2 h) exposed an AUC of 0.632 (95% CI = 0.583–0.682). The Youden’s J index revealed a similar performance for an ISAR cutoff ≥2 and ISAR cutoff ≥3. However, sensitivity of the ISAR cutoff ≥3 was low, which is undesirable for a screening tool (Fig. 2). For the ISAR cutoff ≥2, the sensitivity was 0.696 with a false positive rate of 0.502.

Since only about one quarter of the total cohort (28.7%) received physiotherapy, we analyzed the predictive value of the ISAR score for receiving physiotherapy (yes/no) which resulted in an AUC of 0.603 (95% CI = 0.550–0.657), again with best performance for the ISAR cutoff ≥2 with a sensitivity of 0.691 and a false positive rate of 0.539.

#### Sensitivity and specificity analyses of different CGA abnormal definitions for the prediction of needs (length of hospital stay, nursing hours and physiotherapy)

The definition of an abnormal CGA in case of impairment of ADL plus one other test of the CGA was present in 40.9% of patients. This definition resulted in the best trade-off between sensitivity and false positive rate for the prediction of an increased length of hospital stay and receiving physiotherapy (Tables 3, 5). Only for the prediction of increased nursing, the definition of impairment of ADL plus impairment of cognition or signs of depression (alternative definition A, present in 33.1% of patients) achieved the best performance (Table 4). The definition of impairment of ADL plus impairment of mobility (alternative definition B, present in 28.5%) achieved the lowest performance for all outcomes (Tables 3, 4 and 5).

**Table 3:** Receiver operating characteristics of different CGA abnormal definitions for the prediction of increased length of hospital stay (≥7 days)

	Sensitivity (true positive rate)	Specificity (true negative rate)	1-Specificity (false positive rate)	Youden’s J (true positive - false positive rate)
Impairment of ADL plus another domain	0.493	0.730	0.270	0.223
Alternative definition A: Impairment of ADL plus cognition impairment or signs of depression	0.415	0.790	0.210	0.205
Alternative definition B: Impairment of ADL plus mobility impairment	0.364	0.818	0.182	0.182

*CGA* comprehensive geriatric assessment

**Table 4:** Receiver operating characteristics of different CGA abnormal definitions for increased nursing per day (≥2 h)

	Sensitivity (true positive rate)	Specificity (true negative rate)	1-Specificity (false positive rate)	Youden’s J (true positive - false positive rate)
Impairment of ADL plus another domain	0.556	0.731	0.269	0.286
Alternative definition A: Impairment of ADL plus cognition impairment or signs of depression	0.741	0.798	0.202	0.539
Alternative definition B: Impairment of ADL plus mobility impairment	0.364	0.818	0.182	0.182

*CGA* comprehensive geriatric assessment

**Table 5:** Receiver operating characteristics of different CGA abnormal definitions for receiving physiotherapy during hospital stay

	Sensitivity (true positive rate)	Specificity (true negative rate)	1-Specificity (false positive rate)	Youden’s J (true positive - false positive rate)
Impairment of ADL plus another domain	0.651	0.730	0.270	0.381
Alternative definition A: Impairment of ADL plus cognition impairment or signs of depression	0.566	0.792	0.208	0.358
Alternative definition B: Impairment of ADL plus mobility impairment	0.457	0.797	0.203	0.254

*CGA* comprehensive geriatric assessment

#### Associations of ISAR and CGA results with health outcome (length of hospital stay, nursing and physiotherapy hours, incident fall and type of discharge)

**Fig. 3:**
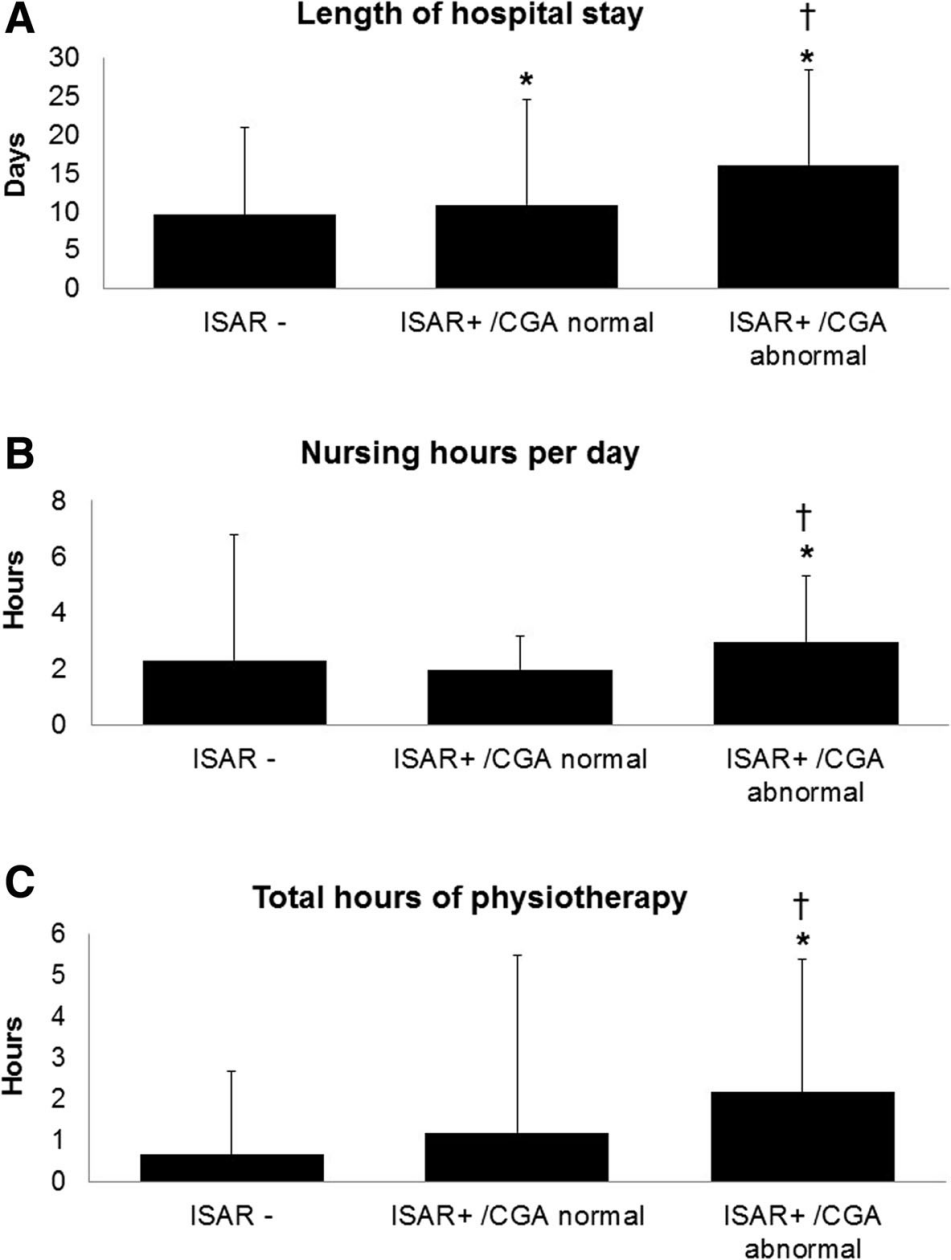
Effects of ISAR and CGA results on length of hospital stay, nursing hours per day and total hours of physiotherapy during hopital stay. CGA, Comprehensive Geriatric Assessment; ISAR, Identification of Seniors at Risk; ISAR+, positive ISAR screening (score ≥ 2); ISAR-, negative ISAR screening (score < 2); CGA abnormal, impairment of ADL plus another domain of the CGA. *p ≤ 0.05 compared to ISAR-, †*p* ≤ 0.05 compared to ISAR+/ CGA normal

Using the ISAR ≥2 cutoff and definition of abnormal CGA as impairment of ADL plus another domain, ISAR+/CGA abnormal patients and ISAR+/CGA normal patients stayed significantly longer in hospital (17.35 ± 18.80 and 10.95 ± 11.85 days) than ISAR- patients (9.60 ± 11.46 days, both comparisons *p* < 0.001, Fig. 3).

ISAR+/CGA abnormal patients also received significantly more hours of nursing care (2.98 ± 2.32) and physiotherapy (2.19 ± 3.19) than ISAR- patients (2.30 ± 4.46 and 0.67 ± 2.02, both p < 0.001) and ISAR+/CGA normal patients (1.97 ± 1.18 and 1.19 ± 4.30, both p < 0.001, Fig. 3).

Incident falls occurred in 4.0% (*n* = 19) of the total cohort with a significantly higher number of falls in ISAR+/CGA abnormal patients (10.1%, *n* = 10) than in ISAR- (2.2%, *n* = 5, *p* = 0.002) and ISAR+/CGA normal (2.8%, *n* = 4, *p* = 0.016) patients.

Although in the total cohort most of the patients terminated their treatment regularly with discharge back home, fewer ISAR+/CGA abnormal patients terminated their treatment regularly with discharge back home (59.6%) compared to ISAR+/ CGA normal (78.5%, p = 0.002) and ISAR- (78.2%, *p* = 0.056) patients (see Table 6).

**Table 6:** Type of hospital discharge in the total internal medicine cohort

	ISAR- (*n* = 229; 48.6%)	ISAR+/CGA normal (*n* = 143; 30.4%)	ISAR+/CGA abnormal (*n* = 99; 21.0%)	*p*-value ISAR+/CGA normal vs ISAR-	*p*-value ISAR+/CGA abnormal vs ISAR-	*p*-value ISAR+/CGA abnormal vs ISAR+/CGA normal
Treatment terminated regularly	179 (78.2%)	113 (78.5%)	59 (59.6%)	0.056	0.133	**0.002**
Treatment terminated regularly, post-treatment planned	34 (14.8%)	17 (11.8%)	19 (19.2%)	0.097	0.715	0.073
Treatment terminated against medical advice	2 (0.9%)	0 (0.0%)	2 (2.0%)	0.999	0.999	0.438
Transfer to another hospital	6 (2.6%)	7 (4.9%)	10 (10.1%)	0.753	0.086	0.280
Death	4 (1.7%)	2 (1.4%)	3 (3.0%)	0.531	0.592	0.190
Discharge to rehabilitation institution	0 (0.0%)	3 (2.1%)	2 (2.0%)	0.137	0.098	0.999
Discharge to nursing institution	0 (0.0%)	0 (0.0%)	2 (2.0%)	0.999	0.315	0.438
Discharge or transfer with subsequent readmission	4 (1.7%)	1 (0.7%)	2 (2.0%)	0.999	0.677	0.999

Data are total numbers complemented in brackets by frequencies. CGA, comprehensive geriatric assessment; ISAR, Identification of Seniors at Risk; ISAR+, positive ISAR screening (score ≥ 2); ISAR-, negative ISAR screening (score < 2); CGA abnormal: impairment of ADL plus another domain of the CGA. Boldface values were significant at *p* <=0.05

## Discussion

While there is a growing interest in understanding the role of geriatric problems for poor health outcomes, there is little information on how geriatric risk screening followed by CGA affects health outcomes of patients hospitalized in internal medicine environments. We demonstrated that abnormal ISAR screening and CGA results were associated with longer hospital stay, more hours of nursing and physiotherapy, higher number of falls and a lower percentage of regularly terminated treatments. In line with previous suggestions [33], an ISAR cutoff ≥2 and the definition of an abnormal CGA as impairment of ADL plus impairment of another CGA domain best predicted patient health outcomes (length of hospital stay, nursing, and physiotherapy hours).

Almost 60% of our patient cohort had a positive ISAR screening, which is comparable to previous studies using similar patient cohorts from emergency departments. In 667 patients aged ≥70 years from emergency departments in the United Kingdom (mean age 80 years), 69% had a positive ISAR screening [34]. In 258 patients aged ≥65 years (mean age: 79 years) from a Canadian emergency department 61.2% screenings were positive [35]. About 40% of our cohort receiving CGA due to positive ISAR screening had an abnormal CGA defined as impairment of ADL plus another domain of the CGA.

In data from the Department of Orthopedics and Trauma Surgery of the University Hospital Essen, we observed a higher proportion of positive ISAR screenings and abnormal CGA results [30]. However, this study revealed similar associations of ISAR and GCA with length of hospital stay and amount of nursing hours indicating suitability of ISAR and CGA in different clinical specialties.

Our ROC results indicated a low discriminating ability of the ISAR tool for length of hospital stay, nursing hours and physiotherapy. This is in line with previous studies demonstrating that ISAR lacks sufficient prognostic validity for various short- and long-term outcomes [7] in contrast to the original development and validation study stating fair performance [36]. A Dutch study including 177 patients aged ≥65 years admitted to internal medicine departments, who were subjected to ISAR, showed sensitivity, specificity and AUC for functional decline measured by self-reported Katz ADL index of 92.9, 39.3% and 0.67, respectively [37, 38]. The different values, specifically for sensitivity, indicate that ISAR may be more suitable for predicting functional decline than length of hospital stay, nursing hours and physiotherapy. Since the present study is based on data available during hospital stay, information about long-term outcomes after the hospital stay including mortality, readmission to hospital or not being able to live at home independently is not available.

We must consider that we applied ISAR screening in internal medicine wards and not in emergency department setting, representing the original and validated setting. Including patients who were already hospitalized, we created a more homogenous patient cohort compared to emergency department setting. In the original studies by McCusker’s group [8, 14, 36], only 35% of the tested emergency department patients were subsequently admitted to the hospital. Further, ISAR was designed for patients aged ≥65 years. In our study cohort inclusion criterion was in most cases an age of ≥75 years, again creating a more homogenous patient group. The low performance of ISAR in ROC analyses could therefore lead to a misclassification which could result in over- or underuse of medical resources. Of course, older patients with higher risk require the provision of a sufficient amount and quality of care which a relevant and indispensable cost factor in hospital and health management [39]. However, since medical resources are valuable but limited, ISAR, alone or combined with CGA, can only be a single element in a process leading to the allocation of patient support.

To the best of our knowledge, this is the first study evaluating the predictive value of ISAR screening and CGA for hospitalized patients’ health outcomes in internal medicine departments. Based on our results, ISAR screening alone may not be suitable for identifying the needs of older hospitalized patients, whereas combination with CGA may allow for the detection of patients requiring longer hospital stay, requiring more hours of nursing and physiotherapy, exhibiting higher risk of falls and having a lower percentage of regularly terminated treatments. Thus, ISAR could help to decide time- and cost-efficiently, which patients should receive a CGA and subsequently be targeted by geriatric interventions [37, 40]. Our findings emphasize that ISAR screenings and CGA should be applied at the time point of a patient’s hospitalization since positive screening and abnormal CGA was associated with more falls during the subsequent hospital stay. The initial use of screening tools is in line with previous recommendations of international geriatric societies [41, 42].

As major limitation, only 41.2% of all eligible patients in our cohort received ISAR screening which, although comparable to other screening implementation trials [35], may not allow truly representative statements for older patient populations, which raises the need for cautious data interpretation. Further limitations are intrinsic to the nature of the ISAR and CGA instruments. It needs to be questioned whether ISAR can reliably be applied to patients with cognitive impairment, which is a common phenomenon in older patient cohorts, since these patients may not answer the ISAR item about memory problems correctly. Furthermore, our CGA did not differentiate the nature of cognitive deficits, which in the setting of acute hospitalized patients may either be related to mild cognitive impairment, dementia or delirious states. Additional influencing factors, such as nutritional status, psychosocial factors or lack of social support, were not assessed. Patients with short hospital stays were under-represented in the cohort receiving ISAR screening followed by CGA. The low ISAR completion rate of 41.2% mirrors some key barriers in the implementation of new screening procedures which requires the compliance of both, patients and staff.

## Conclusions

Abnormal geriatric risk screening and assessment are associated with longer hospital stay and higher amount of nursing and physiotherapy during hospital stay, greater risk of falling, and a lower percentage of successfully terminated treatment in older in-patients. An ISAR cutoff ≥2 and the definition of an abnormal CGA as impairment of ADL plus impairment of another CGA domain best predicted patient health outcomes in our study. Further efforts are urgently needed to optimize geriatric patient management. By increasing the awareness of health professionals, we should be able to establish improved health support procedures that may prevent unfavorable patient outcomes.

## Data Availability

All relevant data are within the paper. If additional data is needed it can be made available from the ethical committee of the University Duisburg-Essen (ethikkommission@uk-essen.de) for researchers who meet the criteria for access to confidential data by contacting the corresponding author.

## Abbreviations

ADL: Activities of daily living
AUC: Area under the curve
CGA: Comprehensive geriatric assessment
CI: Confidence interval
GDS: Geriatric Depression Scale
ISAR: Identification of Seniors at Risk screening
ISAR-: ISAR screening negative < 2
ISAR+: ISAR screening positive ≥ 2
ISAR+/CGA abnormal: ISAR screening positive ≥ 2, but abnormal results in CGA
ISAR+/CGA normal: ISAR screening positive ≥ 2, but normal results in CGA
MMSE: Mini Mental State Examination
SD: Standard deviation
SPSS 22: Statistical Packing for Social Science 22
